# Adverse drug reactions among major depressive disorders: patterns by age and gender

**DOI:** 10.1016/j.heliyon.2021.e08655

**Published:** 2021-12-22

**Authors:** Tariku Sisay, Roza Wami

**Affiliations:** a Department of Biomedical Sciences, College of Health Sciences, Mizan Tepi University, Mizan, Ethiopia; b Department of Clinical Pharmacy, Rift Valley University, Addis Ababa University, Ethiopia

**Keywords:** Adverse drug reaction, Major depressive disorder, Depression, Age, Gender, Antidepressants

## Abstract

**Background:**

Although a significant crisis of adverse drug reaction (ADR) among major depressive disorders (MDDs) is not uncommon, research in Ethiopia has been limited. As a result, the goal of this study was to estimate the prevalence rate of ADRs among MDD patients by age and gender at the outpatient department of Amanuel mental specialized hospital (AMSH) in Addis Ababa, Ethiopia.

**Method:**

The study was conducted on 129 (61 men and 68 women) volunteers at the outpatient department of AMSH, Addis Ababa, Ethiopia, from November 2020–March 2021. A longitudinal cross-sectional study design was employed. All participants were between 35 and 72 years old, with a mean age of 49.5 (SD = 18.8). Patients who had been taking an antidepressant for at least one month and had a follow-up within the first three months after diagnosis and treatment initiation were included in the study. Antidepressant-related ADRs were assessed using the Naranjo ADR probability scale. Antidepressant side-effect checklist (ASEC) as all ADRs have been listed on it was also used to classify a mental state examination (MSE) into mild, moderate, and severe.

**Results:**

According to this study, the overall prevalence of antidepressant-related adverse reactions among MDD patients was 69%, with females having a higher prevalence rate. One of the study's unexpected findings was that ADR was significantly (p = 0.039) higher in young study subjects than in the elderly (73.1% versus 66.2%, respectively). ADRs were shown to be substantially more common in patients taking polypharmacy than in mono-pharmacy (72.5% versus 65%, respectively). The bulk of the ADRs reported were likely, moderate, and probably avoidable. The most common adverse effects reported by patients in the current study were weight gain in TCAs, followed by sexual dysfunction with SSRIs, nausea or vomiting in MAOIs, and headache in SNRIs. The prevalence of ADRs was higher in MAOIs (80%), while SSRIs had the lowest (62.5%). The prevalence of ADRs varies depending on comorbidities: 62.7 % in the absence of comorbidities versus 74.3% in the presence of comorbidities (those with one or more comorbidities).

**Conclusion:**

ADRs that occur in MDD patients are considerable, and gender and age are associated with their occurrence. These findings underscore the importance of monitoring ADRs in mental outpatients frequently to recognize and decrease the risks posed by ADRs earlier. As a result, the quality of care may increase, total health care expenses may decrease, and adherence among patients with depression may improve.

## 1 Introduction

Depending on the severity and pattern of depressive episodes over time, healthcare providers may recommend antidepressant medication as one therapeutic approach. Antidepressant medication, however, has possible adverse effects (WHO, 2018). Adverse drug reactions (ADRs) refer to any unexpected, unintended, undesired, or excessive response to a drug; which occurs at doses normally used in man for prophylaxis, diagnosis, or therapy of disease or the modification of physiologic function (WHO, 2004).

In a nutshell, an ADR is an unpleasant effect that is reasonably associated with the use of medicine, which predicts hazards from future administration and necessitates preventive, particular therapy, dose regimen changes, or product withdrawal. But, ADR is not the same as overdosing or drug maladministration, which can happen by accident or on purpose. The most commonly reported antidepressant ADRs are drowsiness, sexual dysfunction, anticholinergic effects, weight gain, memory and concentration issues, cardiovascular, gastrointestinal, and adverse effects on the metabolism (Ayano, 2016; Peluso et al., 2012; Onder et al., 2003).

Antidepressant treatment studies involve three distinct phases (Mauri et al., 2005); acute, continuation, and maintenance or prophylaxis. The acute phase lasts 2–3 months and necessitates symptom stabilization. The continuation phase, which lasts 3–4 months, is designed to avoid recurrence. In patients at high risk of recurrence, prophylaxis is recommended, with duration of at least several years. In this regard, ADRs can even happen at regular doses used in the acute and maintenance phases of treatment (Sengupta et al., 2011). ADRs affect about 20% of the ambulatory population receiving medications (Downs, 1993). These outpatient occurrences do not always necessitate hospitalization; however, their quality of life would be adversely affected often with varying magnitudes. ADR in its most severe form can cause morbidity, death, and hospital admissions (Angamo et al., 2016).

Data on the burden of ADRs in resource-constrained settings such as Ethiopia are scarce. It is expected that the burden will be even greater due to a variety of attributes such as widespread poor labeling and off-label use; use of herbal remedies and related adverse outcomes and interactions; genetic factors; and nutrition status (Nwokike, 2008). As well, there are insufficient data on drug use practices in several parts of the world, notably Ethiopia.

Professional prescriptions for psychotropic medicines that are routinely prescribed such as antidepressants frequently deviate from established clinical guidelines (Verdoux and Bégaud, 2004; Donoghue and Hylan, 2001). In Ethiopia, psychotropic medicines can be prescribed by primary care doctors in any condition, while there are some restrictions for primary care nurses (WHO, 2006). Even though second-generation pharmacotherapy was introduced in the medical management of depressive disorders decades ago, first-line pharmacologic therapy is still widely used in low-and middle-income countries such as Ethiopia (Ayano, 2016; Sayers, 2001). Although the efficacy of first- and second-generation antidepressants is similar in general, first-generation antidepressants have been reported with many adverse effects. But then second-generation antidepressants with their low side-effect profile have been playing an important role in the treatment of MDD patients (Gartlehner et al., 2011).

In this regard, the American Psychiatric Association (APA) in 2010 (Gelenberg et al., 2010) and the American College of Physicians (ACP) in 2016 (Qaseem et al., 2016) released guidelines for professional prescriptions of psychotropic medicines for MDD patients. According to the guidelines, there is equal efficacy within and between pharmacologic classes; thus, adverse event profiles, patient preferences, dose regimens, prices, and drug interactions should all be taken into account when selecting a medication. In the majority of patients, the guidelines suggest that selective serotonin reuptake inhibitors (SSRIs), serotonin and norepinephrine reuptake inhibitors (SNRIs), bupropion, or mirtazapine is the best first-line treatments. Although tricyclic antidepressants (TCAs) and monoamine oxidase inhibitors (MAOIs) are pharmacologic groups that can be used to treat depression, they are not regarded first-line due to safety concerns and pharmacological characteristics (e.g., drug-drug interactions, complex dosing, and dietary restrictions).

Studies indicate that incorrect perceptions of ADRs, the lack of an effective and well-established pharmacovigilance system can lead to under-reporting of ADRs (Khan et al., 2013). For instance, a study done on physicians' experiences reporting ADRs in Addis Ababa, noticed a gap between ADR cases obtained from medical records and data provided to the national medicine regulatory authority (FMHACA, 2014). Nevertheless, the history of ADR monitoring can be traced back to the widely publicized thalidomide disaster, which ushered in a new era of drug control in many countries. Ethiopia is the 88^th^ country to join a global drug monitoring network of 96 countries (The Uppsala Monitoring Center, Sweden) (Lindquist, 2008).

The WHO guidelines seek countries with the best reporting rates produce more than 200 reports per million people every year. A country with a population of about 110 million people (i.e., Ethiopia) should expect to receive at least 22,000 reports per year. Unfortunately, only 114 reports per year were sent to the global database of individual case safety reports (ICSRs) in the year 2010/11 (Angamo et al., 2016). Overall, Africa is acknowledged to have several known constraints on drug safety monitoring, such as underreporting, inadequate information, and a lack of the denominators for ADR reports (Ampadu et al., 2016).

Many hospitals, however, have created comprehensive programs that lay the groundwork for monitoring and reporting adverse responses, as well as a notification strategy to detect subsequent issues. Pharmacovigilance Program of countries is in charge of identification, assessment, undertaking ADR monitoring efforts, and prevention of ADRs to medicines. The effectiveness of these programs depends on health care professionals’ willingness and commitment to reporting the circumstance to the regulatory body. Despite the fact that Ethiopia has had voluntary ADR reporting since 2002, there has been little or no effort made to evaluate how the monitoring system works in terms of ADR case identification and actions taken to improve it (Ermias et al., 2011; Nadew et al., 2020).

While a major ADRs crisis is not unprecedented, we are aware of very few previous studies in Ethiopia. To aid health care practitioners in developing a plan for dealing with or controlling ADR and its symptoms, a greater understanding of ADR, reporting patterns, and reported sex discrepancies is required. As a result, the purpose of this article was to estimate the prevalence rate of ADRs among MDD patients by age and gender at the outpatient department of AMSH in Addis Ababa, Ethiopia.

## 2 Materials and methods

### 2.1 Ethical statement

This study conducted following the guidelines laid down in the Declaration of Helsinki and approved by the Institutional Ethics Committee of Rift Valley University Department of Clinical Pharmacy. Participants provided written consent to participate, with an explanation of procedures, and risks and benefits in the study.

### 2.2 Consent for publication

We the authors of this research give our consent for publication of identifiable details within the text to be published in the above journal and Article. Informed consent to publish these identifiable images or information has been obtained from the participants.

### 2.3 Subjects

The study was conducted on 129 (61 men and 68 women) volunteers at the outpatient department of AMSH, Addis Ababa, Ethiopia, from November 2020–March 2021. All participants were between 35 and 72 years old, with a mean age of 49.5 (SD = 18.8).

### 2.4 Eligibility

Our source population consisted of all depressed patients who had regular visits to the outpatient department of AMSH in Addis Ababa, Ethiopia. Patients who had been taking an antidepressant for at least one month and had a follow-up within the first three months after diagnosis and treatment initiation were included in the study. But, smokers, pregnant women, and seriously ill individuals (participants who received assistance with daily needs and activities (e.g., personal care, mobility, household activities, transportation, or medically oriented tasks)) were excluded.

### 2.5 Assessments and measures

This study was carried out using a longitudinal cross-sectional study design and purposive sampling techniques. Clinical psychiatrists collected data from individuals who had regular visits to the outpatient Department of AMSH, using a pretested self-administered questionnaire. Antidepressant-related ADRs were assessed using the Naranjo ADR probability scale, which relied on the interval (9–12). A sum greater than nine was empirically defined as “definitely” having caused the ADR; a sum of five to eight “probably” caused the ADR; a sum of one to four “possibly” caused the ADR; and a score less than one indicated an association with the drug was “doubtful” (Naranjo et al., 1981). Antidepressant side-effect checklist (ASEC) as all ADRs have been listed in it was also used to classify a mental state examination (MSE) into mild, moderate, and severe (Uher et al., 2009). The data collector was trained intensively on the contents of the questionnaire, data collection methods, and ethical concerns. Patients were requested to report only the most annoying adverse effect of a given antidepressant during the last month.

### 2.6 Statistics analysis

Analyses were performed using the Statistical Package for Social Sciences (SPSS) (version 21.0; IBM, Armonk). Categorical variables are presented as the frequency and percentage, whereas numerical variables were presented as the means and SDs. To assess baseline demographics, clinical features, suspected drugs, severity, and type of ADR, descriptive statistical analyses were conducted. Categorical variables were analyzed using chi-square. A p-value <0.05 was considered significant.

## 3 Results

### 3.1 Characteristics of study participants

A total of 129 (61 males and 68 females) patients with MDD, participated in this study. The study was conducted from November 2020 to March 2021 at the outpatient department of AMSH, Addis Ababa, Ethiopia. The age range of the study subjects ranges from 35 to 72 years, with individuals aged 55 and up constituting the largest group of study subjects (59.7%). The most commonly prescribed antidepressant category among 129 patients receiving antidepressants was TCAs (33.3%), followed by SSRIs (24.8%) (Table 1).

**Table 1:** Socio-demographic characteristics of MDD patients at the Outpatient Department of Amanuel Mental Specialty Hospital from November 2020 to March 2021 (n = 129).

Characteristics	Variables	Frequency	Percent
Age	35–55 years	52	40.3
	>55 years	77	59.7
Sex	Female	68	52.7
	Male	61	47.3
	Can't read &write	42	32.5
Educational status	Primary education	53	41
	Secondary education	21	16.2
	Higher education	13	10
Comorbidity	No disease	59	45.7
	Only one disease	65	50.4
	More than one disease	5	3.9
	SNRIs	24	18.6
Treatments	SSRIs	32	24.8
	TCAs	43	33.3
	MAOIs	30	23.2
	Mono-pharmacy	60	46.5
Medication	Polypharmacy	69	53.5

TCAs: tricyclic antidepressants; SNRIs: serotonin-norepinephrine reuptake inhibitors; SSRIs: selective serotonin reuptake inhibitors; MAOIs: monoamine oxide inhibitors.

### 3.2 Prevalence of ADR and associated factors

The prevalence of ADR among MDD patients was 89 (69%). The prevalence of ADRs in women and men, respectively, is 70.6% versus 67.2%. One of the study's unexpected findings was that ADR was significantly (p = 0.039) higher in young study subjects than in the elderly (73.1% versus 66.2%, respectively). ADRs were shown to be substantially more common in patients taking polypharmacy than in mono-pharmacy (72.5% versus 65%, respectively). The prevalence of ADRs varies depending on comorbidities: 62.7 % in the absence of comorbidities versus 74.3% in the presence of comorbidities (those with one or more comorbidities) (Table 2).

**Table 2:** Displays the prevalence rate of ADRs by gender, age, therapy, and the presence or absence of comorbidities (n = 129).

Variables		Frequency (%)	p-value
Gender	Male	41 (67.2)	
	Female	48 (70.6)	0.041
Comorbidities	No disease	37 (62.7)	
	One or more disease	52 (74.3)	0.047
Age	35–55 years	38 (73.1)	
	>55 years	51 (66.2)	0.039
Medication	Mono-pharmacy	39 (65)	
	Polypharmacy	50 (72.5)	0.018

A p-value <0.05 was considered significant.

**Figure 1:**
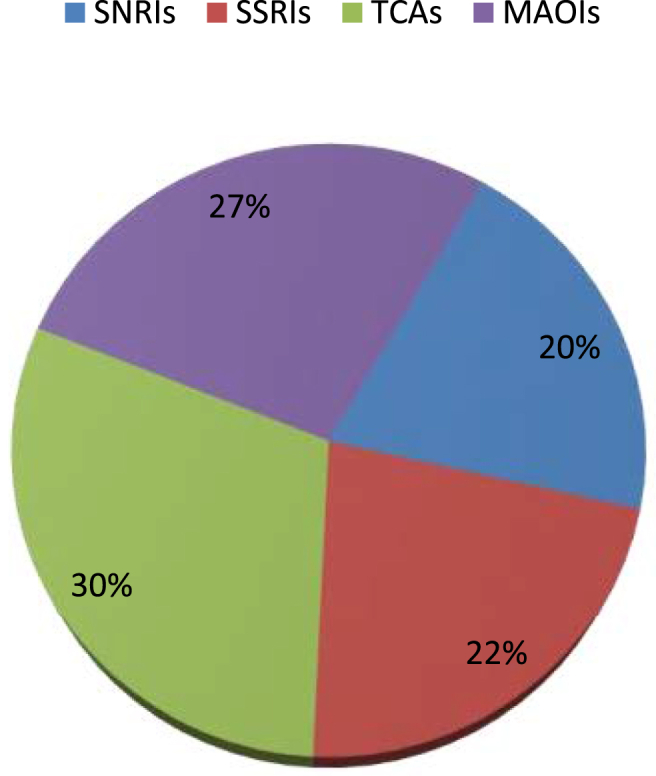
Shows the percentage of each antidepressant category in the participants with ADRs TCAs: tricyclic antidepressants; SNRIs: serotonin-norepinephrine reuptake inhibitors; SSRIs: selective serotonin reuptake inhibitors; MAOIs: monoamine oxide inhibitors.

**Figure 2:**
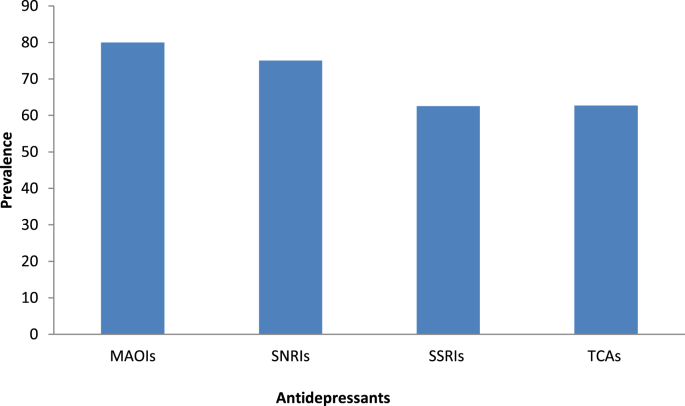
Shows the prevalence of ADRs by type of antidepressant.

In terms of severity, 28% of respondents have had a mild, 50% have had a moderate, and 21% have had severe ADRs. The most common adverse effect reported by patients were weight gain (74%) in TCAs, followed by sexual dysfunction (70%) in SSRIs, nausea or vomiting (66.7%) in MAOIs, and headache (61.1%) in SNRIs (Table 3). Moreover, TCAs had the highest proportion of ADRs (30%), while SNRIs had the lowest (20%) (Figure 1). Furthermore, the prevalence of ADRs was higher in MAOIs (80%), while SSRIs had the lowest (62.5%) (Figure 2).

**Table 3:** Indicates the prevalence of the most commonly mentioned adverse effects by patients in each antidepressant category.

Adverse effects	SNRIs (n = 18)	SSRIs (n = 20)	TCAs (n = 27)	MAOIs (n = 24)
	Frequency (%)	Frequency (%)	Frequency (%)	Frequency (%)
Blurred vision	3 (16.7)	0	7 (25.9)	6 (25)
Diarrhea	0	4 (20)	3 (11.1)	2 (8.3)
Disorientation	0	0	3 (11.1)	0
Drowsiness	5 (27.8)	12 (60)	6 (22.2)	10 (41.7)
Headache	11 (61.1)	5 (25)	15 (55.6)	11 (45.8)
Nausea or vomiting	3 (16.7)	3 (15)	16 (59.3)	16 (66.7)
Problem with urination	5 (27.8)	2 (10)	4 (14.8)	3 (12.5)
Sexual dysfunction	8 (44.4)	14 (70)	0	7 (29.2)
Tremor	6 (33.3)	9 (45)	9 (33.3)	4 (16.7)
Weight gain	7 (38.9)	10 (50)	20 (74.1)	9 (37.5)

TCAs: tricyclic antidepressants; SNRIs: serotonin-norepinephrine reuptake inhibitors; SSRIs: selective serotonin reuptake inhibitors; MAOIs: monoamine oxide inhibitors.

## 4 Discussion

ADRs can be caused by any medicine, although not all patients have the same level or type of reaction to the same treatment regimen. A better understanding of ADRs, reporting patterns, and reported sex discrepancies are needed to prevent future ADR-related negative impacts. As a result, the goal of this study was to determine the prevalence of ADRs in MDD patients by age and gender at the outpatient department of AMSH in Addis Ababa, Ethiopia. In this perspective, some of the study's notable findings in this area are as follows.

### 4.1 ADRs and MDD

ADR has been a prevalent complication with antidepressants. A study done on MDD patients in Ethiopia by Abegaz et al. (2017) reported that more than eighty percent of respondents had ADRs. In line with this, our study found that over 69% of MDD patients experienced ADR. However, these ADRs cases would not be reported due to a lack of motivation and commitment on the part of health providers, inaccurate conceptions about ADRs, and a lack of an effective and well-established Pharmacovigilance system.

Many medical schools including those in Ethiopia should include Pharmacovigilance in their undergraduate and postgraduate curriculums to have an efficient Pharmacovigilance program. A study of knowledge, attitudes, and behaviors may give information on the factors that contribute to underreporting of ADR. As a result, increasing public knowledge of Pharmacovigilance should be the first step toward making the reporting process more straightforward.

In terms of the severity of the ADR, the current study found that half of the participants had a moderate adverse reaction. ‘Moderate ADRs’ are those that require a change in drug therapy, a specific treatment, or an increase in hospitalization of at least one day (Jones, 1982). According to studies, the severity of ADRs, rather than the occurrence of ADRs, was a significant determinant in the poor prognosis of therapy outcomes (Liao et al., 2013). Moreover, the most commonly prescribed antidepressant was TCAs (specifically amitriptyline), followed by the SSRIs fluoxetine. The finding is consistent with another study in Ethiopia (Dugassa et al., 2018). The reasons for this result could be due to the low cost, prescribers may regularly prescribe earlier generation antidepressants (which have a wide variety of side effects) in Ethiopia. Prescription studies are critical in determining drug usage patterns in health care settings. It also identifies areas where corrective actions could be taken to improve therapy and better health outcomes.

Moreover, the most common adverse effect reported by patients in the current study was weight gain in TCAs, followed by sexual dysfunction in SSRIs, nausea or vomiting in MAOIs, and headache in SNRIs. Treatment settings for patients with MDD need to identify the least restrictive setting (e.g., continuum of possible levels of care, from involuntary hospitalizations to partial hospital programs, skilled nursing homes, and in-home care) that is most likely to address safety and achieve improvement in the patient's condition is crucial when prescribing antidepressants. We also need to consider the patient's clinical status, such as symptom severity, co-occurring mental or general medical disorders, and level of functioning; available support systems; and ability to effectively care for oneself, provide reliable feedback to the psychiatrist, and participate in therapy.

### 4.2 ADRs and age ranges

ADRs can be caused by any medicine, although not all patients have the same level or type. Findings have indicated that older patients with age >55 years were markedly associated with the prevalence of ADRs (Pirmohamed et al., 2004; Kongkaew et al., 2008; Beijer and De Blaey, 2002). This could be because the increased susceptibility to ADRs seen in the elderly may be associated with distribution, metabolism, and excretion, which are all heavily dependent on age (Ermias et al., 2011; Schurig et al., 2018).

Moreover, as individuals grow older, the amount of water in the body reduces while fat tissue grows. As a result, water-soluble drugs concentrate at higher levels. Drugs with such extending effects affect the volume of distribution, which might raise the risk of toxicity or ADR (Klotz, 2009). Furthermore, lower albumin levels in older individuals, which can be caused by chronic illness, malnutrition, disease, or drug-related anorexia, and lower oral intake due to poorly fitting dentures, might result in higher free or active drug fractions, which in turn can increase the risk of adverse effects.

However, an intriguing finding of this study was that the prevalence of ADR is significantly higher in younger individuals than in the elderly. This could be because an individual's physiological characteristics appear to be more important than chronological age in determining whether or not a patient would tolerate a given drug (Ozcan et al., 2016).

### 4.3 ADRs and polypharmacy

The literature varies in its definition of polypharmacy; however, the underlying concept of taking more drugs at the same time than is clinically indicated remains consistent. According to WHO, rational drug usage demands that patients receive drugs that are appropriate for their medical condition, in doses that fit their specific needs, for a defined period, and at the lowest feasible cost to them and their community (Acurcio et al., 2004). Although there is no concrete definition of the term, it has come to mean the use of several (usually five or more) medications on a daily basis, with the possibility that not all of these are clinically necessary. Polypharmacy is more likely to occur as a result of the number of prescriptions recommended for concurrent comorbidities; nevertheless, this does not mean that patients should not take any medications. In this study, ADR was significantly associated with polypharmacy, which is consistent with previous studies (Beijer and De Blaey, 2002; Pirmohamed et al., 2004; Budnitz et al., 2011).

One of the significant factors responsible for the growth of ADRs from polypharmacy is related to the incapacity of certain patients, particularly the elderly, to keep track of when and how they take their drugs, regardless of how well they perform when taken alone. Patients who are not strict enough about taking their prescriptions as recommended will drop out of therapy and fail to take their medications appropriately (Alomar, 2014).

There are hardly any studies that suggest a link between a person's educational standing and their failure to take their prescriptions as prescribed, which could be related to health literacy, the patient's capacity to interpret instructions, and the knowledge they should gain about their disease and therapy. In the current study, 41% of participants had primary education, while 32.5 % were unable to read or write. This could potentially play a role in the high occurrence of ADR.

In this perspective, to prevent the early occurrence of ADRs, it should be important to educate the patient and, where appropriate, his or her family using language that the patient can read and understand; clarifying frequent misunderstandings about the condition (e.g., depression is not a real illness) and therapy (e.g., depression is not a real sickness) (e.g., antidepressants are addictive). And also it would be worthy to educate patients on the importance of completing a full course of treatment, the danger of relapse, and the early detection of repeated symptoms.

Chumney and Robinson (2006) have provided evidence that the probability of ADRs due to polypharmacy is estimated at 13% when two concurrent medications are taken, rising to 50% for four medications and 100% for seven or more medications prescribed simultaneously. As a result, determining the prevalence rate of ADRs due to polypharmacy among MDD patients would facilitate the development of guidelines and policies for this vulnerable population.

### 4.4 ADRs and gender

Women have historically been excluded from clinical trials due to concerns about the impact of shifting hormones of data, and researchers on both human and animal subjects still do not adequately account for gender differences in data. Sex-based dose modifications were developed only after decades of post-marketing complaints of cognitive problems in women given the usual male dose (Zucker and Prendergast, 2020). Women are approximately twice as likely as males experience ADRs across all drug classes, and they are nearly twice as likely to be hospitalized as a result of an ADR (Tharpe, 2011; Nakagawa and Kajiwara, 2015). Consistently, the prevalence of ADR in the current study was found considerably higher in females than male counterparts. Women have been shown to have more polypharmacy experiences than men, and they use more different prescriptions per year, which may contribute to female ADRs (Manteuffel et al., 2014), but also emphasizes the significance of sex-aware dosing. Sex may be a substantial risk factor for ADRs for a variety of biological, psychological, and societal reasons. In general, this could be due to physiological differences between males and females that might alter pharmacokinetics (i.e., the induction or inhibition of metabolizing enzymes) and pharmacodynamics (i.e., additive or antagonistic pharmacological effects) of drugs (Meyer et al., 2009).

## 5 Limitations and strengths

This study had several limitations, including a lack of randomization, self-reported data, and a short intervention period, all of which could limit the study's generalizability to others. . Indeed, the study revealed the magnitude of ADR in MDD with gender and age, which can inform future research on community-based mental health.

## 6 Conclusion

According to this study, the overall prevalence of antidepressant-related adverse reactions among MDD patients was 69%, with females having a higher prevalence rate. The bulk of the ADRs reported were likely, moderate, and probably avoidable. The most commonly prescribed antidepressant was TCAs (specifically amitriptyline), followed by the SSRIs fluoxetine. The most common adverse effect reported by patients were weight gain in TCAs, followed by sexual dysfunction with SSRIs, nausea or vomiting in MAOIs, and headache in SNRIs. These findings underscore the importance of clinical pharmacists monitoring ADRs in mental outpatients frequently to recognize and decrease the risks posed by ADRs earlier. As a result, the quality of care may increase, total health care expenses may decrease, and adherence among patients with depression may improve.

## Declarations

### Author contribution statement

Tariku Sisay and Roza Wami: Conceived and designed the experiments; Performed the experiments; Analyzed and interpreted the data; Contributed reagents, materials, analysis tools or data; Wrote the paper.

### Funding statement

This research did not receive any specific grant from funding agencies in the public, commercial, or not-for-profit sectors.

### Data availability statement

Data included in article/supplementary material/referenced in article.

### Declaration of interests statement

The authors declare no conflict of interest.

### Additional information

No additional information is available for this paper.
